# Conference Report: SIXTH BIENNIAL SYMPOSIUM ON OPTICAL FIBER MEASUREMENTS Boulder, CO September 11–12, 1990

**DOI:** 10.6028/jres.096.012

**Published:** 1991

**Authors:** G. W. Day, D. L. Franzen

**Affiliations:** Electromagnetic Technology Division, National Institute of Standards and Technology, Boulder, CO 80303

The development of lightwave communications over the last 2 decades has been particularly dependent on careful metrology to support product specification and standardization. Some estimates have suggested that as much as 20% of the selling price of a fiber is associated with measurements. This perhaps explains the continuing success of the biennial Symposium on Optical Fiber Measurements as an international forum for the discussion of lightwave measurement problems.

The sixth in this series of symposia sponsored by the National Institute of Standards and Technology (NIST) in cooperation with the Institute of Electrical and Electronics Engineers (IEEE) and the Optical Society of America (OSA) was held September 11-12, 1990 at NIST in Boulder. The 2 day meeting drew about 250 attendees; 30 percent came from 16 countries outside of the United States. In addition, several committees of the International Electrotechnical Commission (lEC) and the Telecommunications Industry Association (TIA) concerned with standards for optical fiber measurements took the opportunity to hold meetings on the day before and the 2 days following the Symposium.

Attendees saw and heard a total of 45 papers, 9 invited and 36 contributed, slightly more than half from abroad. As a result of the large number of papers submitted, the committee elected for the first time to schedule a poster session.

The topics of contributed papers at the Symposium have historically been a good measure of current problems facing the industry. At the 1990 meeting there were enough papers on each of three topics to require separate sessions.

One of those was geometric measurements on single mode fiber. Maintaining a high degree of circularity in the cladding of a fiber, an accurate cladding diameter, and good concentricity between the core and cladding are essential in achieving low connector losses. Current measurement agreement for cladding diameter as determined by international comparisons is about 0.4 µm (1 standard deviation). For manufacturers to further reduce geometric tolerances on fiber, primary standards and measurement methods with uncertainties approaching 0.1 µm are needed. To achieve that accuracy it has become necessary to revisit the capabilities of existing methods and to investigate new approaches.

Keith Emig of Corning, Inc. presented data indicating that transverse interferometric techniques, based on Fizeau and Michelson interferometers, could achieve the required 0.1 µm accuracies in cladding diameter measurements. He regards these techniques as suitable for measuring samples to be used for calibrating measurement systems based on microscopic end-viewing. End-viewing is an easy and popular technique, but suffers from difficulty in edge definition. A team from the National Physical Laboratory (NPL) in England described their experiments with various metal-on-glass artifact standards for the calibration of end-viewing measurement systems and reported that with sufficient care, calibration to the required level can be achieved. However, Matt Young, of NIST, reported systematic errors between 0.1 and 0.2 µm using metal-on-glass standards for calibration. He also reported difficulties in contact micrometer methods related to deformations of the fiber by the measurement device. Scanning confocal microscopy, in which the object is illuminated “point by point by a laser, as the object scans past the focal point of a microscope,” is also being investigated at NIST in the hope that it can avoid edge definition problems.

A second area requiring a full session was measurements in integrated optics. Guide attenuation and effective indices remain important problems. Workers at the Centro Studi e Laboratori Telecommunicazioni (CSELT), in Italy, reported that it is possible to measure both of these parameters simultaneously by examining the Fabry-Perot fringes of the cavity formed by the guide while scanning the optical frequency. A paper from the University of Florida described another method of obtaining mode indices, based on a Mach-Zehnder interferometer. Workers at NIST demonstrated that the photothermal deflection technique can be used to obtain loss measurements over an extremely large dynamic range: from less than 0.5 dB/cm in an ordinary waveguide, to 760 dB/cm in a high quality polarizer. There were, in addition, several papers on the characterization of integrated optic modulators.

The third single-topic session was devoted to various techniques of reflectometry. Optical time domain reflectometry has, for 15 years, been essentially the only nondestructive method for obtaining measurements of any parameter in the fiber as a function of length. Now, other versions of reflectometry are being investigated. These include frequency domain techniques and white light interferometric techniques. The motivation for this work is better parameter estimation and, especially, better spatial resolution. In some cases, spatial resolutions on the order of a micrometer are contemplated.

A particularly interesting new approach to obtaining distributed strain measurements, reported in an invited paper from the Nippon Telegraph and Telephone Transmission Systems Laboratories (NTT-Ibaraki) is Brillouin optical time domain reflectometry. This technique uses the fact that the Brillouin shifted lines are stress dependent, and has been successfully used for *in situ* measurements of the strain in undersea cables.

A Technical Digest containing summaries of all of the papers presented at the Symposium is available from the Superintendent of Documents, U.S. Government Printing Office, Washington, DC 20402. Order NIST Special Publication 792, September 1990, stock number SN-003-003-03025-2. The price is $11.00.

